# Nuclear iASPP may facilitate prostate cancer progression

**DOI:** 10.1038/cddis.2014.442

**Published:** 2014-10-23

**Authors:** E V Morris, L Cerundolo, M Lu, C Verrill, F Fritzsche, M J White, G N Thalmann, C S ten Donkelaar, I Ratnayaka, V Salter, F C Hamdy, X Lu, R J Bryant

**Affiliations:** 1 Ludwig Institute for Cancer Research Ltd, University of Oxford, Nuffield Department of Clinical Medicine, Old Road Campus Research Building (off Roosevelt Drive), Headington, Oxford, UK; 2 Nuffield Department of Surgical Sciences, University of Oxford, Old Road Campus Research Building (off Roosevelt Drive), Headington, Oxford, UK; 3 Department of Cellular Pathology and NIHR Oxford Biomedical Research Centre, Oxford University Hospitals NHS Trust, John Radcliffe Hospital, Headley Way, Oxford, UK; 4 Department of Urology, Inselspital, Bern, Switzerland

## Abstract

One of the major challenges in prostate cancer (PCa) research is the identification of key players that control the progression of primary cancers to invasive and metastatic disease. The majority of metastatic PCa express wild-type p53, whereas loss of p63 expression, a p53 family member, is a common event. Here we identify inhibitor of apoptosis-stimulating protein of p53 (iASPP), a common cellular regulator of p53 and p63, as an important player of PCa progression. Detailed analysis of the prostate epithelium of iASPP transgenic mice, iASPP^Δ8/Δ8^ mice, revealed that iASPP deficiency resulted in a reduction in the number of p63 expressing basal epithelial cells compared with that seen in wild-type mice. Nuclear and cytoplasmic iASPP expression was greater in PCa samples compared with benign epithelium. Importantly nuclear iASPP associated with p53 accumulation *in vitro* and *in vivo.* A pair of isogenic primary and metastatic PCa cell lines revealed that nuclear iASPP is enriched in the highly metastatic PCa cells. Nuclear iASPP is often detected in PCa cells located at the invasive leading edge *in vivo*. Increased iASPP expression associated with metastatic disease and PCa-specific death in a clinical cohort with long-term follow-up. These results suggest that iASPP function is required to maintain the expression of p63 in normal basal prostate epithelium, and nuclear iASPP may inactivate p53 function and facilitate PCa progression. Thus iASPP expression may act as a predictive marker of PCa progression.

Prostate cancer (PCa) is the commonest male malignancy and second leading cause of male cancer-related death in the Western world. As metastatic PCa can kill patients it is important to understand the molecular switches responsible for progression of localised disease to an invasive and metastatic phenotype.

Mutation of the tumour suppressor gene p53, one of the commonest mutated genes in human cancers,^1^ is associated with invasive metastatic PCa. p53 mutation or loss-of-heterozygosity rates in early PCa are low,^2, 3, 4, 5, 6^ suggesting p53 mutation is a late event in PCa and that selection for loss of p53 function occurs during PCa progression.^7, 8, 9, 10, 11, 12, 13, 14, 15^ Mutant p53 can induce cancer cell invasion by binding to p63 and inhibiting its transcriptional activity.^16, 17, 18, 19^ p63 is a member of the p53 family and shares high sequence similarity to p53 at its DNA binding domain. p63 is predominantly expressed in basal epithelial cells and is a master transcription factor that determines epithelial stratification. There are six p63 isoforms and TAp63*α*, *β* and *γ* contain different lengths of C terminus, whereas ΔNp63*α*, ΔNp63*β* and ΔNp63*γ* lack the N-terminal transactivation domain. p63 is expressed in the basal cells of normal adult prostate epithelium^20^ with ΔNp63*α* being the most prominent isoform.^21^ ΔNp63-positive cells of the urogenital sinus can generate all prostate epithelial cell lineages in mice,^22^ suggesting that these are stem/progenitor cells. Loss of the p63-expressing basal cell layer is a hallmark of invasive PCa.^21,23,24^ Mutant p53 is mainly detected in metastatic PCa cells, therefore it is unlikely that mutant p53 induces PCa cellular invasiveness by inhibiting p63 function. In addition, as p53 mutations or copy number loss are detected in only 25%^2^ of metastatic PCa, it is likely that p53 loses its tumour suppressor function in metastatic disease through other means. Cellular regulators of p53 may be responsible for the inactivation of p53.

Increased expression of two p53 inhibitors, mouse double minute 2 homologue and inhibitor of apoptosis-stimulating protein of p53 (iASPP), is responsible for the inactivation of wild-type p53 in human malignant melanoma^25^ which, like PCa, has a low rate of p53 mutation. iASPP belongs to the ASPP family of proteins that comprise iASPP, ASPP1 and ASPP2. ASPP1 and ASPP2 were originally identified as activators, and iASPP as an inhibitor, of p53-mediated apoptosis.^26,27^ Studies have demonstrated that ASPP2 is a haploinsufficient tumour suppressor,^28, 29, 30^ and ASPP1 and ASPP2 cooperate with oncogenic RAS to potentiate RAS signalling.^31, 32, 33^ ASPP2 can exert its tumour suppressor function by mediating RAS-induced cellular senescence and by inhibiting RAS-induced autophagy in primary cells.^31,33^ ASPP2 also increases RAS-induced p53-mediated transcription and apoptosis in cancer cells.^32^ In normal epithelial cells, ASPP2 can bind and colocalise with protease activated receptor 3 thereby maintaining the integrity of cell polarity and adherence junctions.^34,35^ ASPP2 is a novel suppressor of squamous cell carcinoma through its ability to repress ΔNp63 expression via a nuclear factor kappa-light-chain-enhancer of activated B cells-mediated pathway.^30^ iASPP is thought to function as an oncoprotein as it is over-expressed in several malignancies^36, 37, 38, 39^ including a small cohort of PCa cases.^40^ Consistent with this, iASPP is highly expressed in basal epithelial cells and its expression level decreases upon cellular differentiation *in vitro* and *in vivo*.^30,41^ iASPP is an inhibitor of cellular senescence and iASPP deficiency accelerates differentiation of keratinocytes *in vitro* and *in vivo*, partly through iASPP's ability to bind and regulate p63 function.^41,42^ These observations are in agreement with *in vivo* findings that iASPP and ASPP2 are key regulators of epithelial stratification with opposing functions, partly through their ability to exert opposing regulation of p63 expression and activity.^30,41,42^ We recently showed that ASPP2 represses ΔNp63 expression^30^ whilst iASPP is known to induce p63 expression in keratinocytes,^42^ and we also showed that iASPP binds p63 and regulates its transcriptional activity to suppress cellular senescence and differentiation.^41^ Since p63 is required for mouse prostate development and its expression is lost in invasive PCa, we investigated whether iASPP plays a role in mouse prostate development through its ability to regulate p63. The potential role of iASPP in regulating the behaviour of p63-negative PCa cells was also investigated in PCa samples.

## Results

### iASPP deficiency causes a reduced number of p63-expressing basal cells and increased expression of differentiation markers

**Figure 1:**
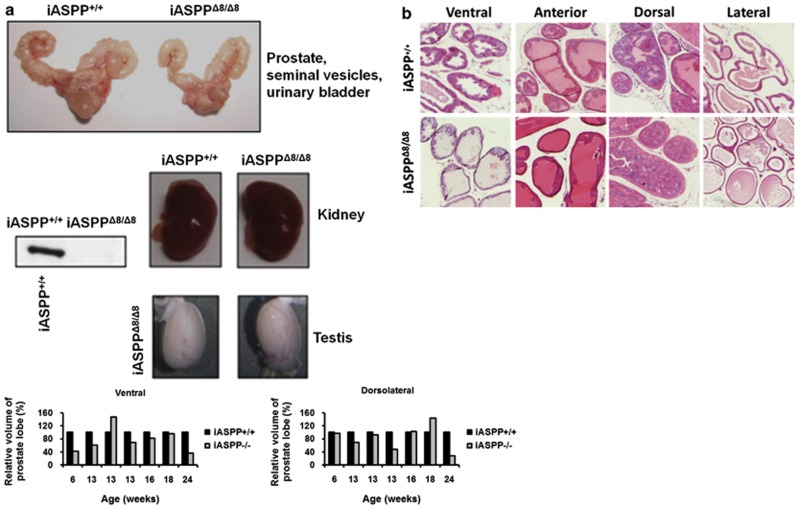

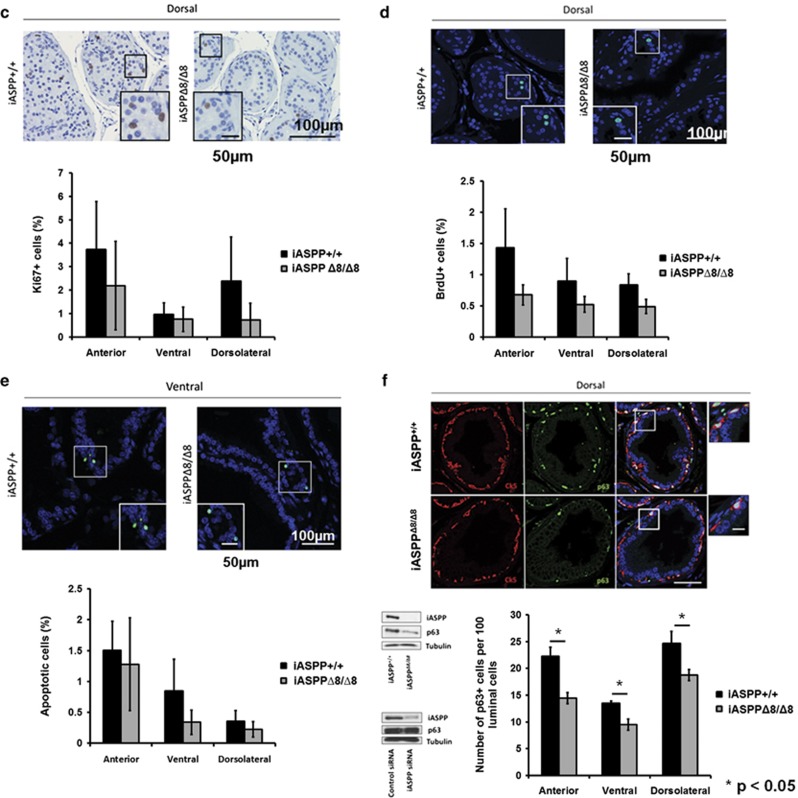
Phenotypic analysis of iASPP^Δ8/Δ8^ mouse prostates. (**a**) The prostate glands of iASPP^Δ8/Δ8^ mice were macroscopically similar to those of wild-type mice. (**b**) iASPP^Δ8/Δ8^ mouse prostate lobes developed with an intact epithelial lining and a lumen. (**c**) iASPP^Δ8/Δ8^ mouse (*n*=3) prostate lobes demonstrated a small reduction in the number of Ki67-positive actively proliferating epithelial cells compared with iASPP^+/+^ (*n*=3) controls. (**d**) iASPP^Δ8/Δ8^ (*n*=4) mice demonstrated a small reduction in the number of BrdU-labelled cells in each lobe of the prostate following BrdU injection compared with iASPP^+/+^ (*n*=3) controls. (**e**) iASPP^Δ8/Δ8^ mice (*n*=5) demonstrated a small reduction in the number of TUNEL-positive apoptotic epithelial cells in each lobe of the prostate compared with iASPP^+/+^ (*n*=5) controls. **(f)** iASPP^Δ8/Δ8^ mice (*n*=5) had significantly fewer p63-positive basal epithelial cells compared with iASPP^+/+^ mice (*n*=5). The expression level of TP63 in prostate protein extracts from iASPP^Δ8/Δ8^ mice was diminished compared with iASPP^+/+^ controls

To investigate the effect of iASPP loss on gross prostate morphology the prostate glands from iASPP^Δ8/Δ8^ mice were analysed macroscopically. The prostate gland lobes of iASPP^Δ8/Δ8^ mice were sometimes smaller than those of age-matched wild-type mice but were not significantly smaller across the cohort (*P*>0.05) (Figure 1a). There was no difference in overall size of these animals. The prostate lobes of iASPP^Δ8/Δ8^ mice had an epithelial cell layer and a lumen with similar haematoxylin and eosin (H&E) appearances to wild-type (Figure 1b). These results suggest that iASPP deficiency is unlikely to grossly affect mouse prostate development.

We showed previously that iASPP inhibits apoptosis and cellular senescence, therefore we tested whether iASPP deficiency enhances apoptosis or inhibits cellular proliferation, and we observed a small reduction in the number of Ki67-expressing cells in iASPP^Δ8/Δ8^ mice compared with wild-type (Figure 1c). iASPP^Δ8/Δ8^ mice demonstrated a small reduction in the number of bromodeoxyuridine (BrdU)-labelled cells in all prostate lobes compared with wild-type (Figure 1d). Similar number of apoptotic prostate epithelial cells were observed in iASPP^Δ8/Δ8^ mice compared with wild-type (Figure 1e). These results suggest that iASPP loss does not enhance apoptosis but reduces cellular proliferation in the mouse prostate.

iASPP has recently been found to be highly expressed in the nucleus of basal cells where it interacts with TP63, and its expression becomes cytoplasmic in differentiated cells.^41,42^ We observed that iASPP was expressed in both the TP63-positive basal cell layer and the TP63-negative luminal cell layer (Supplementary Figure 1A) of mouse prostate epithelium. Both cytoplasmic and nuclear iASPP expression accords with previous studies.^43^ The iASPP staining is specific and is confirmed by absence of signal in iASPP^Δ8/Δ8^ mice (Supplementary Figure 1B). We observed a significant decrease in the number of p63-expressing epithelial cells in all lobes of iASPP^Δ8/Δ8^ mouse prostates compared with wild-type (Figure 1f).

Since p63-expressing cells are progenitors and iASPP deficiency is known to facilitate keratinocyte differentiation, the effects of loss of iASPP function on prostate epithelial cell differentiation were investigated. The prostate mainly consists of basal and luminal cells, and a small subpopulation of neuroendocrine cells.^44^ Although the basal and luminal cells are discrete populations, intermediate cells express a mixture of basal and luminal markers^45, 46, 47, 48^ and are thought to have entered the differentiation programme and be transitional between basal and luminal phenotypes. The cytokeratin (Ck) expression pattern can be used to distinguish between these three populations of cells. In prostate epithelium, basal cells express Ck5 and Ck14, luminal cells express Ck8 and Ck18 and intermediate cells express basal and luminal Cks and Ck19. Basal and luminal cells were present in iASPP-deficient mice as demonstrated by Ck5 and Ck8 IF (Supplementary Figure 1C). To determine the levels of Ck expression, protein was extracted from prostate tissue from three iASPP^Δ8/Δ8^ and three age-matched wild-type mice. We observed an increase in Ck19 expression in iASPP^Δ8/Δ8^ compared with wild-type prostate (Supplementary Figure 1D) with minimal changes in Ck5 and Ck8 expression. These results suggest that iASPP deficiency facilitates p63-expressing progenitors to commit to the luminal cell linage by transitioning into intermediate cells, and that iASPP regulates mouse prostate development through its ability to inhibit cellular senescence and prostate epithelium differentiation. The findings that iASPP is expressed in both basal and luminal prostate epithelial cells suggest that iASPP may affect prostate epithelial cell functions through p63-dependent and -independent pathways.

### iASPP is expressed in human prostate epithelium and nuclear iASPP associates with invasive phenotypes

**Figure 2:**
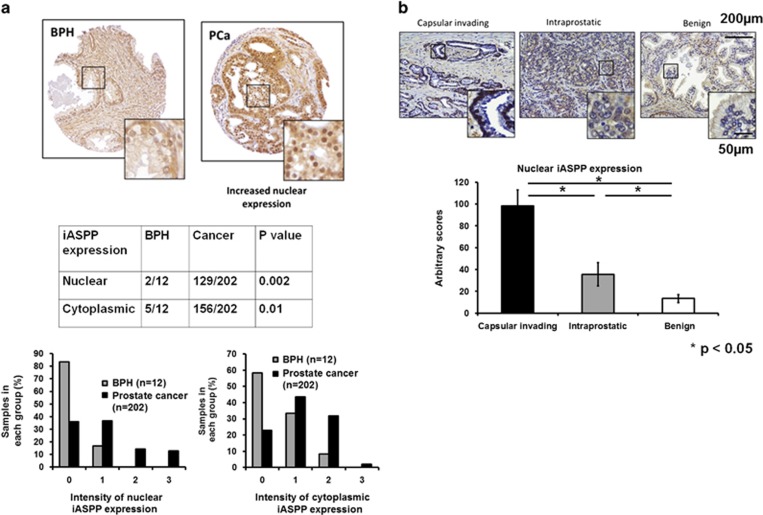
iASPP expression differs between benign and malignant human prostate epithelium. (**a**) iASPP is mainly expressed in the nucleus and cytoplasm of p63-positive basal cells, and to a lesser extent in the nucleus of luminal epithelial cells, in benign human prostate epithelium. Nuclear (*P*=0.002) and cytoplasmic (*P*=0.01) iASPP expression is increased in human prostate cancer samples compared with benign epithelial cell samples. **(b)** Nuclear iASPP expression was greater in prostate cancer cells invading through the capsule in locally invasive pT3a disease compared with areas of intra-prostatic tumour or benign prostate epithelium

To investigate whether iASPP plays a role in human PCa we first examined iASPP expression pattern in a number of benign prostate samples using an anti-iASPP mouse monoclonal antibody LX049.3^41^ to carry out IHC staining. We observed that iASPP was predominantly expressed in the nucleus and cytoplasm of p63-positive basal cells in benign prostate epithelium. Occasional low levels of iASPP expression were also observed in benign luminal epithelial cells (Figure 2a). We noticed that the benign epithelium adjacent to areas of PCa expressed significantly greater levels of iASPP than benign prostate epithelium samples taken from men without PCa (Supplementary Figure 2A). Consistent with this an increase in both cytoplasmic and nuclear iASPP expression was observed in PCa samples in comparison to benign prostate epithelium (Figure 2a). By analysing iASPP expression in PCa cells with different invasive properties within locally advanced cases, we observed that nuclear (Figure 2b) and cytoplasmic (Supplementary Figure 2B) iASPP expression was significantly greater in cells at the ‘leading edge' invading the prostate capsule compared with cancer cells within intra-prostatic tumour. These results suggest that iASPP expression patterns differ in PCa cells. High levels of nuclear and cytoplasmic iASPP associates with PCa cells with an invasive property, whereas low iASPP-expressing PCa cells are confined to the gland and are not invading locally.

### Slow migrating nuclear iASPP is enriched in metastatic PCa cells

**Figure 3:**
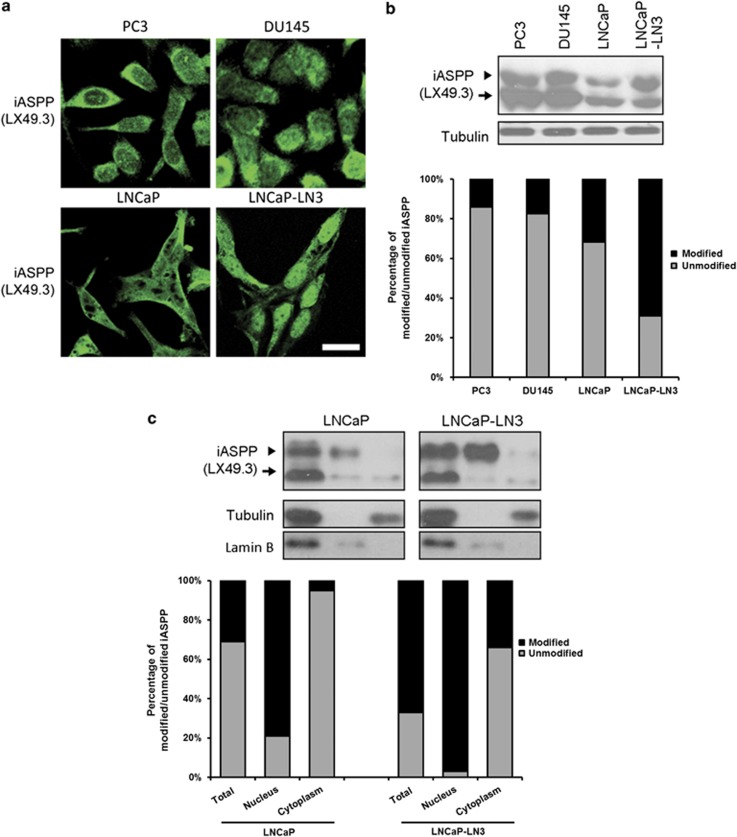
Phosphorylated iASPP accumulates in the nucleus of prostate cancer cells. (**a**) Highly metastatic LNCaP-LN3 prostate cancer cells expressed predominantly nuclear iASPP, whereas LNCaP cells with lower metastatic potential expressed iASPP with a relatively equal distribution between nucleus and cytoplasm. The p53-null PC3 cell line and p53-mutant (P233L and V274F) DU145 cell line expressed predominantly cytoplasmic iASPP. (**b**) iASPP migrated as two bands in an immunoblot of whole-cell protein extracts from prostate cancer cells. LNCaP-LN3 cells predominantly expressed the slower migrating iASPP band previously identified as being nuclear localised phosphorylated iASPP. LNCaP cells expressed both slow- and fast-migrating iASPP, corresponding with its roughly equal subcellular distribution. PC3 and DU145 cells, which exhibited predominantly cytoplasmic localised iASPP, expressed predominantly fast-migrating unmodified cytoplasmic iASPP. (**c**) The slower migrating modified iASPP band is primarily detected in nuclear protein extracts, whereas the faster migrating unmodified iASPP band is localised primarily to the cytoplasm. Invasive metastatic LNCaP-LN3 cells express a greater proportion of modified nuclear iASPP than non-invasive LNCaP cells

N-terminal phosphorylation of iASPP retards its migration in sodium dodecyl sulfate (SDS) gel and causes nuclear accumulation in cells,^25^ and nuclear iASPP localisation is associated with human melanoma metastasis.^25^ The observation that nuclear iASPP is enriched in PCa cells with invasive properties led us to test the hypothesis that iASPP phosphorylation may be enhanced in metastatic PCa cells *in vitro* using a pair of isogenic LNCaP cell lines. LNCaP-LN3 is a metastatic derivative of LNCaP cells. Using IF we observed that p53-null PC3 cells and p53-mutant (P233L and V274F) DU145 cells^49^ expressed predominantly cytoplasmic iASPP. In wild-type p53-expressing LNCaP cells, iASPP is expressed with a relatively equal distribution between nucleus and cytoplasm, whereas LNCaP-LN3 derivatives expressed predominantly nuclear iASPP (Figure 3a). iASPP migrated as two bands in WBs of these PCa cell lines however the proportion of slow-migrating iASPP was highest in LNCaP-LN3 cells corresponding with the enrichment of modified nuclear iASPP in these cells. LNCaP cells expressed both slow- and fast-migrating iASPP with similar intensity and this expression pattern correlates with the roughly equal cellular distribution of iASPP detected in these cells. PC3 and DU145 cells expressed predominantly fast-migrating unmodified cytoplasmic iASPP (Figure 3b). The slower migrating modified iASPP band is primarily detected in nuclear protein extracts, whereas the faster migrating unmodified iASPP band is localised primarily to the cytoplasm (Figure 3c). LNCaP-LN3 cells expressed a greater proportion of modified nuclear iASPP than LNCaP cells. These results illustrate that modified nuclear iASPP is enriched in PCa cells with high metastatic potential.

### Increased nuclear iASPP expression correlates with TP53 accumulation *in vivo* and in organotypical co-cultures *in vitro*

**Figure 4:**
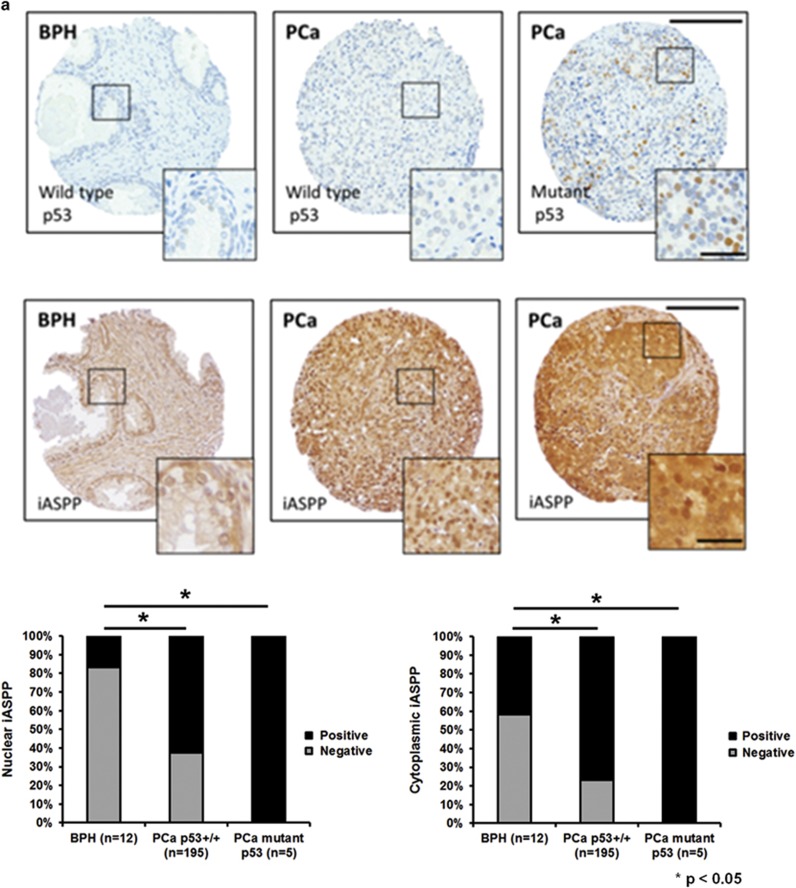

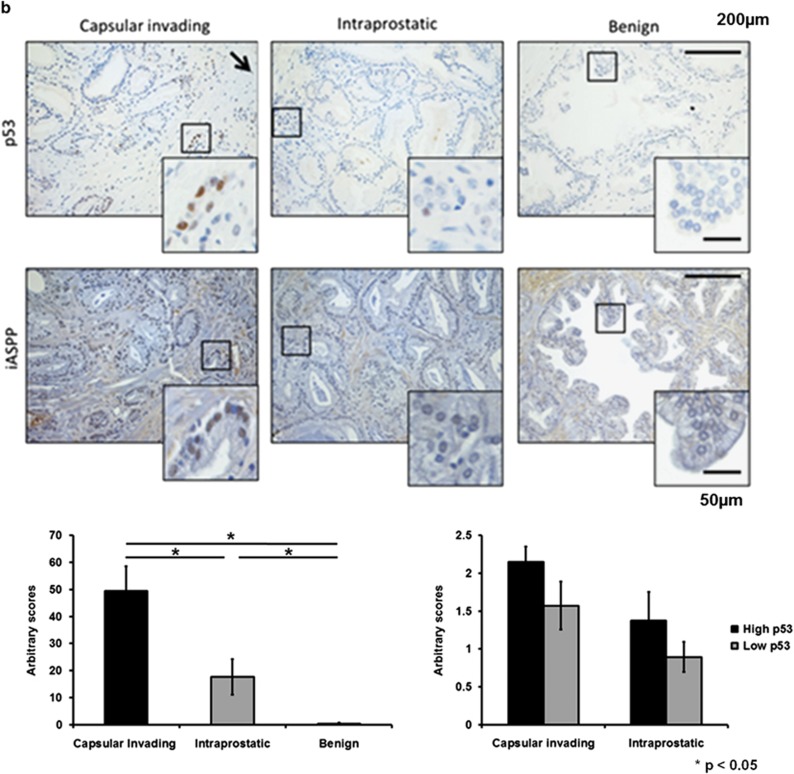

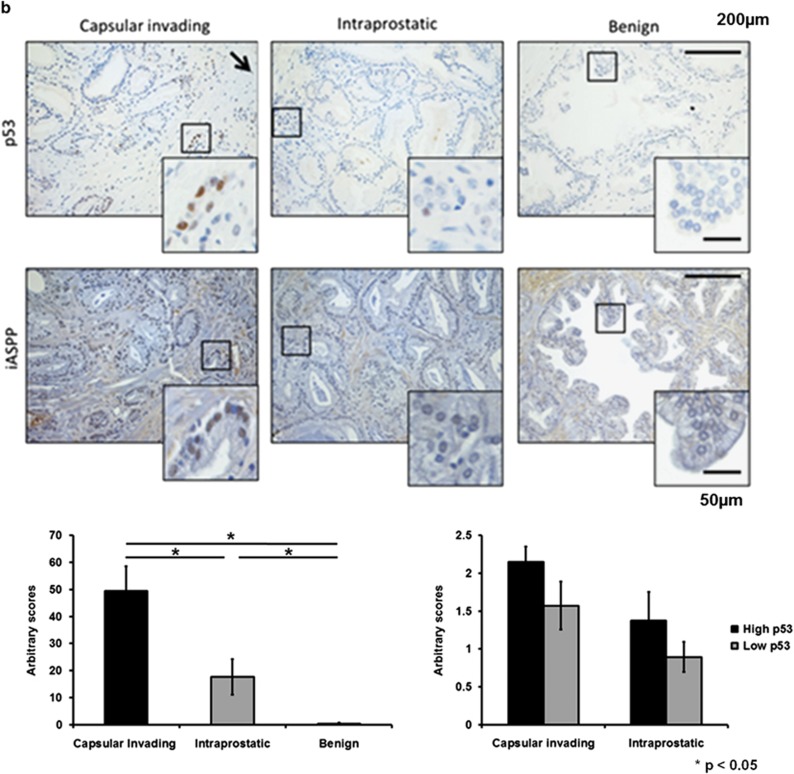
Nuclear iASPP expression was increased in prostate cancer samples with a high level of TP53 expression. (**a)** iASPP expression in human prostate cancer samples was greater in both wild-type p53 (<30% nuclei expressing TP53) and mutant p53 (>30% nuclei expressing TP53) samples compared with benign prostate epithelium (*P*<0.05). Although the number of mutant p53 prostate cancer samples was small (*n*=5) compared with the number of wild-type p53 samples (*n*=195) we observed that the nuclear iASPP expression was higher in mutant p53 samples compared with wild-type p53 samples. (**b**) TP53 and iASPP expression in cells within the leading edge of locally invading pT3a prostate cancer was compared with other areas of the sample. TP53 expression was greatest in invading prostate cancer cells within the leading edge. iASPP expression was higher in prostate cancer cells with high TP53 expression, compared with cancer cells with low TP53 expression, in capsular-invading and intra-prostatic areas of tumour. Arrow depicts direction of invasion into the prostate capsule. (**c**) Nuclear iASPP is co-expressed with nuclear TP53 in DU145 prostate cancer cells at the invasive margin in organotypic co-cultures. **(d)** Immunoprecipitation of TP53 probed with anti-iASPP and anti-TP53 antibodies demonstrated that iASPP interacts with mutant TP53

p53 mutation is a late event in PCa progression. High levels of TP53 expression is often associated with *p53* mutation and is detected in invasive metastatic PCa cells. In our tissue microarray (TMA) cohort of PCa samples we only detected high levels of TP53 expression (>30% of cancer cell nuclei^14^) in 5/200 PCa samples. We observed that nuclear iASPP expression was significantly higher in samples strongly expressing TP53 compared with samples with absent or weak TP53 expression (Figure 4a). High levels of TP53 expression were detected within cells at the ‘leading edge' of locally invasive PCa compared with intraprostatic tumour cells. Higher levels of nuclear TP53 expression were detected in locally advanced PCa cases compared with organ-confined tumours (Figure 4b). These results suggest a potential association between high levels of TP53 and nuclear iASPP accumulation in the leading edge of locally invasive PCa.

This hypothesis was tested by performing an *in vitro* organotypic co-culture using DU145 cells known to harbour mutant *p53*^49^ and express high levels of TP53, which accumulates in the nucleus. Using double IF staining we observed that some DU145 cells with accumulated TP53 also expressed nuclear iASPP (Figure 4c). DU145 cells with co-localisation of iASPP and TP53 were preferentially seen at the ‘leading edge' of the layer of cells within the organotypic co-cultures. This contrasts with observations in organotypic co-cultures of PC3 cells which do not express TP53, and LNCaP and LNCaP-LN3 cells expressing wild-type TP53. The association between nuclear TP53 and nuclear iASPP accumulation in organotypical co-cultures of DU145 cells suggests that mutant TP53 may interact with nuclear iASPP. Using an immunoprecipitation assay we observed that an anti-iASPP antibody was able to co-immuno-precipitate TP53 with iASPP in DU145 cell lysates (Figure 4d). These results suggest an association between the accumulation of mutant TP53 and nuclear iASPP, and that mutant TP53 is able to bind iASPP *in vitro*.

### Increased iASPP expression in PCa is associated with an adverse clinical outcome

**Figure 5:**
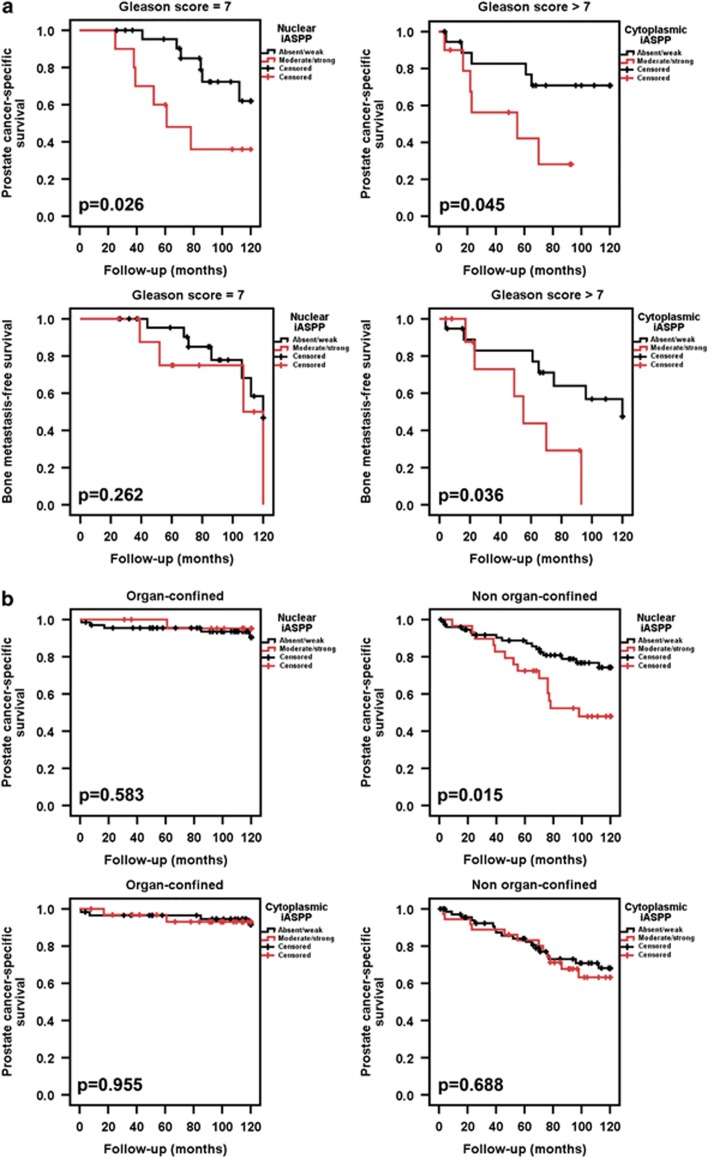
Increased iASPP expression in prostate cancer samples is associated with an adverse clinical prognosis. (**a)** Increased nuclear iASPP expression in intermediate grade (Gleason sum score 7) prostate cancers was associated with increased prostate cancer-specific death after 10 years of follow-up following radical surgery. Increased cytoplasmic iASPP in high grade (Gleason sum score ≥8) prostate cancer samples was associated with an increased risk of both prostate cancer bone metastasis development and prostate cancer-specific death after 10 years of follow-up following radical surgery. (**b**) Increased nuclear iASPP expression was associated with a poor clinical outcome in men treated surgically for non organ-confined (≥pT3a) prostate cancer. High nuclear iASPP in this group of patients was associated with an increased risk of prostate cancer-specific death following radical surgery

We investigated whether the increased iASPP expression observed in PCa samples might have prognostic clinical significance using a TMA of samples from 203 patients undergoing radical prostatectomy with prolonged clinical follow-up^50^ (Table 1). Increased iASPP expression was associated with an adverse clinical outcome. Increased nuclear iASPP expression in intermediate grade (Gleason sum score 7) PCa was significantly associated with PCa-specific death. Increased cytoplasmic iASPP expression in high grade (Gleason sum score ≥8) PCa was associated with both metastasis formation and PCa-specific death (Figure 5a). The prognostic significance of tumour stage, pre-operative prostate-specific antigen level, and nuclear and cytoplasmic iASPP expression were tested in a Cox regression multivariate analysis (Table 2). Nuclear iASPP expression in Gleason 7 tumours was prognostic for PCa-specific death, whilst cytoplasmic iASPP in high-grade (Gleason ≥8) tumours was prognostic for the development of bone metastases and PCa-specific death.

Increased nuclear iASPP expression was associated with an increased risk of PCa-specific death in patients with locally advanced PCa undergoing radical prostatectomy, whereas this association was not seen in organ-confined disease (Figure 5b). These results suggest that increased expression of nuclear and cytoplasmic iASPP has clinical significance by conferring an increased risk of developing metastases or resultant PCa-specific death following treatment of early disease.

## Discussion

The transcription factor p63 is specifically expressed in prostate basal epithelial cells^21,51, 52, 53^ and altered p63 expression is utilised in PCa diagnosis.^20,21,23,54,55^ A ‘Prostate-63 Cancer Diagnostic Test' has been approved by the US Food and Drug Administration (FDA) as a diagnostic marker of PCa in clinical samples. In agreement with the importance of p63 in the tumorigenesis of PCa, transgenic mouse studies have established a crucial role for p63 in the development of the mouse prostate gland. We show here for the first time that iASPP, a cellular regulator of p63, also plays a role in the normal development of the mouse prostate gland. We observed a detectable reduction in the number of proliferating cells in iASPP^Δ8/Δ8^ mouse prostate compared with wild-type mice. Interestingly, we observed significantly fewer TP63-expressing basal cells within the prostate epithelium of iASPP^Δ8/Δ8^ compared with wild-type mice, suggesting iASPP may be required to maintain the TP63-positive basal cell layer. Consistent with a role for iASPP in suppressing epithelial differentiation, we observed an increase in the expression level of Ck19, a marker of intermediate prostate epithelial cells,^47,56^ in iASPP^Δ8/Δ8^ mouse prostates. Cells which are phenotypically intermediate between basal and secretory cells have been found to be enriched in proliferative inflammatory atrophy lesions of the prostate, suggesting that these cells may be implicit in PCa.^57^ Our observations in iASPP^Δ8/Δ8^ mouse prostate epithelium are in complete agreement with previous findings whereby iASPP inhibits cellular senescence and binds and regulates p63 function in maintaining homeostasis of stratified epithelium in mouse skin and oesophagus.^41,42^ An increased differentiation process caused by iASPP deletion may accelerate the rate of commitment of p63-expressing progenitors to trans-differentiate into luminal epithelial cells via intermediate cells. In turn this could result in an enhanced diminishment of prostate progenitors.

We observed that loss of iASPP function results in a reduced number of p63 expressing basal cells in prostate epithelium. It has recently been shown that iASPP can regulate p63 expression via a microRNA autoregulatory feedback loop.^42^ It has also been demonstrated that iASPP binds p63 and directly regulates its activity. Importantly iASPP is able to inhibit cellular senescence and epithelial cell differentiation.^41^

It is possible that absence of iASPP induces basal prostate epithelial cells to undergo differentiation due to iASPP's ability to regulate p63 activity and maintain the proliferative potential of basal epithelial cells. Future studies are needed to understand how iASPP deficiency causes a reduction in p63 expressing prostate epithelial basal cells. Regardless of this our observation that nuclear iASPP expression was increased in PCa progression was interesting given that the expression of TP63 is frequently lost in PCa.^24^ It is known that p63 expression defines the basal epithelial cell lineage, and we speculate that when PCa cells enter their metastatic phase there is possible cellular reprogramming, lineage change and an epithelial-to-mesenchymal transition (EMT). p63 is known to regulate key epithelial proteins such as cadherins and cell adhesion molecules.^58^ Moreover, p53 is an important inhibitor of EMT^59^ and most PCa express wild-type p53.^2, 3, 4, 5, 6^ Therefore an inhibitor of p53 such as iASPP could play an important role in facilitating prostate epithelial cells to undergo EMT. We showed recently that nuclear iASPP has the highest p53 binding and p53 inhibition potency.^25^ It is perhaps via this mechanism that nuclear iASPP contributes to loss of p53 function, induction of EMT, loss of the epithelial cell lineage and concomitant loss of p63 expression. This hypothesis warrants further investigation.

The observation that iASPP is predominantly expressed in the nucleus, and to a lesser degree in the cytoplasm of basal prostate epithelial cells in benign prostate samples, suggests that iASPP may maintain the proliferative potential of basal epithelial cells. The finding that iASPP co-localises with TP63 within the nucleus of benign prostate epithelial cells agrees with previous findings where iASPP binds and directly regulates TP63 function.^41^ We observed that iASPP expression was increased in the nucleus and cytoplasm of PCa cells in a cohort of over 200 PCa samples compared with benign prostate epithelial samples. This agrees with an oncogenic role for iASPP in PCa development. It is also in agreement with numerous findings showing an increase in iASPP expression in human cervical, head and neck, ovarian, melanoma and a small cohort of PCa samples.^25,39,40,60,61^ To date, however, changes in iASPP expression have not been associated with long-term prognosis in PCa. Our detailed analysis of iASPP expression patterns in a cohort of 61 PCa samples containing benign prostate epithelial cells adjacent to areas of cancer allowed us for the first time to obtain evidence of a potential ‘field change effect' of iASPP expression. iASPP expression levels in the histologically normal prostate directly adjacent to cancer were seen to be significantly greater than benign epithelium in men without PCa. This observation is in keeping with other published reports, suggesting that a ‘field change' phenomenon or ‘field cancerisation' effect exists during malignant transformation of prostate epithelium.^62, 63, 64, 65, 66, 67, 68, 69, 70^ Furthermore we observed that nuclear iASPP is enriched within PCa cells at the invasive front of locally invading tumours *in vivo*, and within the experimentally derived metastatic PCa cell line LNCaP-LN3 compared with parental LNCaP cells *in vitro*. Interestingly, LNCaP cells contain wild-type p53 and the majority of human PCa also express wild-type p53 with this gene only mutated or deleted in ~30% of the cases. Our findings concur with those in a recent study showing a nuclear enrichment of iASPP in malignant metastatic human melanoma cells,^25^ and this is interesting given that both melanoma and PCa generally have a lower rate of p53 mutation than many other cancer types.

We observed that cytoplasmic iASPP expression, in addition to nuclear iASPP, is detected in PCa samples, and increased cytoplasmic iASPP was associated with an increased risk of both metastasis formation and PCa-specific death following radical surgery. We do not currently understand the biological importance of cytoplasmic iASPP, however p53 has previously been detected in the cytoplasm as well as the nucleus of cells, and levels of cytoplasmic iASPP expression have been associated with an adverse prognosis in other cancer types.^39^ It is possible that cytoplasmic iASPP may inhibit the activity of cytoplasmic p53, although it is known that nuclear iASPP has a higher binding affinity to p53.^25^ Cytoplasmic iASPP may influence aspects of p53 function independent of its transcriptional activity, however further studies will be necessary to fully elucidate the biological role of cytoplasmic iASPP.

These findings suggest that changes in iASPP expression and subcellular localisation may potentially be useful clinically as a biomarker predictive of clinically aggressive PCa. The results of this study identify iASPP as being a key molecular switch in the progression of PCa to an invasive and metastatic phenotype with a lethal outcome.

## Materials and Methods

### Mice

The generation of iASPP^Δ8/Δ8^ mice has been described previously.^41^ All animal procedures were approved by local ethical review and licensed by the UK Home Office. All iASPP^Δ8/Δ8^ mice analysed in this study were paired with age-matched wild-type controls and these were littermates wherever possible. Prostate tissues were fixed in 10% buffered formalin overnight, dehydrated in an ethanol series and cleared in xylene before being embedded in paraffin. The entire prostate was embedded as a whole in a consistent manner to avoid problems with changes in orientation of the lobes. Four micrometre sections were cut for thickness for experimental analysis. For cell proliferation studies, mice were injected with 30 *μ*g BrdU per gram of body weight 24 h before being killed.

### Cell lines

LNCaP (May 2003), PC-3 (February 2004), and DU145 (December 2011) cells were purchased from ATCC (Manassas, VA, USA). LNCaP-LN3 cells were a generous gift from Professor Curtis Pettaway (Department of Urology, The University of Texas MD Anderson Cancer Center, Houston, TX, USA). These cells were maintained in RPMI 1640 medium supplemented with 10% fetal calf serum (FCS) (GE Healthcare Life Sciences, Little Chalfont, UK), sodium pyruvate, MEM vitamins and MEM non-essential amino acids (Invitrogen, Paisley, UK) and were used for no longer than 6 months post resuscitation. Normal human fibroblast (NHF) cells were maintained in Dulbecco's Modified Eagle Medium (DMEM) (Invitrogen) supplemented with 10% FCS. All cell lines were supplemented with 100 U/ml of penicillin and streptomycin (Invitrogen) and incubated at 37 °C in a 5% CO_2_ atmosphere.

### Organotypic culture

A suspension of 5 × 10^5^ NHFs was prepared in DMEM for each organotypic culture. One millilitre scaffold gel plugs were prepared for each organotypic culture containing 5 × 10^5^ NHFs, 10% FCS and 1 : 1 rat tail collagen type I/Matrigel (Becton Dickinson, Oxford, UK) in DMEM, and after overnight incubation at 37 °C, 5 × 10^5^ PCa cells (PC3, DU145, LNCaP and LNCaP-LN3) were plated onto the upper surface of each gel plug and incubated for a further 24 h. A sterile nylon square in a Petri dish was covered with 250 *μ*l gel containing 10% FCS, 70% collagen and DMEM and polymerised by incubating at 37 °C for 20 min. Ten millilitre 1% glutaraldehyde (Sigma, Gillingham, UK) in phosphate buffered saline (PBS) was then added to the Petri dish and the nylon sheets incubated at 4 °C for 1 h then washed in PBS followed by DMEM and then incubated at 4 °C overnight. The nylon sheets were then mounted on metal scaffolds and placed in a Petri dish, and the NHF/PCa cell-containing gel plugs were placed onto the nylon sheet and incubated at 37 °C for 10 days with culture media just beneath the nylon sheet. Gels were then formalin-fixed, processed and sectioned for histology.

### Protein extracts

Protein extracts were obtained from cultured cells or homogenised mouse prostate using urea buffer. Mouse prostate lysates were sonicated prior to protein quantification. Protein concentrations were determined using a protein assay reagent system (Bio-Rad, Hercules, CA, USA) and spectrophotometric analysis at 595 nm. The absorbance of samples was compared with a standard curve derived from known concentrations of bovine serum albumin (BSA; Sigma).

### Antibodies

Primary antibodies used in this study for Western blotting (WB), immunofluorescence (IF), and immunohistochemistry (IHC) include: rat anti-BrdU (ab6326, IF 1 : 200); rabbit anti-Ck 19 (ab53119, WB 1 : 500); rabbit anti-Ck 5 (ab53121, WB 1 : 750, IF 1 : 3000); rabbit anti-Ck 8 (ab59400, WB 1:500, IF 1 : 200); mouse anti-lamin B (ab8983, WB 1 : 1000); rabbit anti-tubulin (ab52901, WB 1 : 2000) (Abcam, Cambridge, UK); mouse anti-iASPP (purified LX049.3, IF 1 : 250, IHC 1 : 250, WB 1 : 1000); rabbit anti-ki67 (Vector Laboratories, Peterborough, UK; IHC 1 : 200); mouse anti-Ku80 (ThermoFisher Scientific, Loughborough, UK; WB 1 : 2000); rabbit anti-p53 (CM1, WB 1 : 1000, IF 1:250); mouse anti-p53 (DO-1, WB 1 : 1000); mouse anti-p63 (4A4, Santa Cruz, Dallas, TX, USA; WB 1 : 1000; IF/IHC 1 : 200).

Secondary antibodies (Invitrogen unless stated) used in this study include: rabbit anti-mouse horseradish peroxidase (HRP) (Dako, Ely, UK; WB 1 : 2000), swine anti-rabbit (Dako, WB 1 : 2000), goat alexa-fluor 488 anti-rabbit (IF 1 : 400), goat alexa-fluor 488 anti-mouse (IF 1 : 400); donkey alexa-fluor 488 anti-mouse (IF 1 : 400); donkey alexa-fluor 647 anti-mouse (IF 1 : 400), donkey alexa-fluor 488 anti-rabbit (IF 1 : 400), donkey alexa-fluor 647 anti-rabbit (IF 1 : 400); goat biotinylated anti-mouse IgG (Vector Laboratories, IHC 1 : 250); goat biotinylated anti-rabbit IgG (Vector Laboratories, IHC 1 : 250).

### Immunoblotting

For whole-cell extracts, cells were lysed in urea buffer containing 8 M urea, 1 M thiourea, 0.5% 3-[(3-cholamidopropyl)dimethylammonio]-2-hydroxy-1-propanesulfonate, 50 mM dithiothreitol and 24 mM spermine. Nuclear and cytoplasmic extract fractions were prepared from 5 × 10^5^ cells using a nuclei isolation kit (NUC101-1KT, Sigma-Aldrich, UK) according to the manufacturer's instructions and the fractions were then directly dissolved in urea buffer. After centrifugation the supernatant was dissolved in SDS loading buffer prior to immunoblot analysis. A total of 100 *μ*g of protein extract was loaded per lane into SDS-polyacrylamide gels. Gels were transferred onto nitrocellulose membrane (Protran, Sigma-Aldrich) and the resulting blots incubated first with primary antibody for 16 h at 4 °C, and then with the appropriate HRP-conjugated secondary antibody (Dako). Protein expression was visualised by enhanced chemiluminescent detection (Amersham Biosciences, Little Chalfont, UK) using X-ray film. All autoradiographs were scanned using a photo scanner. For the detection of specific iASPP isoforms extracts were immunoblotted on a 16 × 12 cm 6% gel. The antibody used for iASPP immunoblotting was LX49.3^41^ unless otherwise stated.

### Immunocytochemistry

Cells grown to 70–80% confluence on coverslips were fixed with 4% paraformaldehyde solution, permeabilised with 0.1% Triton X solution, blocked with 0.2% BSA and primary antibody was added for 1 h at room temperature. Secondary antibody and 4′,6-diamidino-2-phenylindole (DAPI) was then added and coverslips were mounted with mowiol-glycerol solution.

### Immunoprecipitation

Cells grown on 15 cm dishes were washed with cold PBS and lysed with 100 mM NaCl, 1 mM ethylenediaminetetraacetic acid (EDTA), 20 mM Tris (NET) 1% NP-40 IP buffer containing protease inhibitors. Cell lysates were centrifuged at 13 200 r.p.m., 4 °C, for 20 min, and the protein concentration of the supernatant determined. Fifty microlitres of each lysate was used as an input control. Protein G sepharose beads were washed in cold PBS, made up to a ratio of 1 : 1 with PBS and stored at 4 °C. Each lysate was pre-cleared using 30 *μ*l of slurry beads and rotation for 60 min at 4 °C, and then lysates were centrifuged at 4 °C. Ten microlitres of antibody was added to the supernatant along with 30 *μ*l of fresh protein G sepharose beads and the sample rotated overnight at 4 °C. Beads were washed in NET IP buffer and 0.2% NP-40 before being centrifuged further. The supernatant was then removed and sample buffer added to the beads. Samples were then boiled, centrifuged and sample buffer added.

### Human tissue samples

TMA sections were obtained from the Department of Urology, University of Bern, Switzerland. Following IHC protein expression, scores for each core were assigned by a histopathologist (FF and VS). Pathologic tumour stage 3 (pT3) PCa whole-mount sections were obtained from the Oxford Centre for Histopathology Research, Nuffield Division of Clinical Laboratory Sciences, University of Oxford, Oxford Radcliffe Hospitals National Health Service (NHS) Trust, Oxford, UK. After IHC protein expression scores for different areas of interest were assigned by a histopathologist (CV). This study had ethics committee approval (reference number 09/H0606/78).

### IHC and IF

Sections were de-paraffinised in histoclear and rehydrated through graded alcohols to water. Endogenous peroxidise activity was inactivated where appropriate using 3% H_2_O_2_ in methanol, and where necessary antigen retrieval was performed using boiling citric acid (human tissue, TP63) or EDTA (mouse tissue, TP63 and iASPP) buffers. Sections were blocked with 5% normal goat serum and incubated with primary antibody at 4 °C overnight. For IHC, a biotinylated secondary antibody was added for 30 min followed by an avidin/biotin-based peroxidase solution and incubated with 3,3′-diaminobenzidine solution before counterstaining with haematoxylin. For IF, an appropriate secondary antibody with DAPI was added for 1 h. All sections were dehydrated using increasing percentages of ethanol followed by histoclear and mounted in mowiol-glycerol medium. The sections were viewed and photographed with a confocal (IF) or light (IHC) microscope. Where described H&E and Alcian blue staining, were performed using standard techniques.

### Tissue apoptosis assay

Apoptotic cells were detected using an ApopTag Red *In Situ* Apoptosis Detection Kit (Merck Millipore, Watford, UK) according to the manufacturer's recommended protocol.

### Quantification of prostate lobe size and protein expression in mouse and human prostate tissue studies

The size of the prostate ventral and dorsolateral lobes was measured based on the assumption that that they are elliptical in shape. Four microlitre sections were cut through the entire prostate and every tenth section stained using H&E. The largest surface area of each lobe section was calculated using Image J software (NIH Image, Bethesda, MD, USA), and the volume of each lobe calculated using the depth of each lobe (taken from the number of sections available) using the equation: lobe volume=(4/3) × π × *r*^1^ × *r*^2^ × *r*^3^ (where *r*^1^=height, *r*^2^=width, *r*^3^=depth).

All cell counts in mouse prostate tissue studies were performed using a minimum of 1000 cells. Four fields of view at × 20 magnification per slide were randomly selected for each prostate lobe. Three different slides were used per analysis and per animal. Analysis was performed using Image J software (NIH Image).

iASPP expression intensity in human tissue samples was scored by a histopathologist (CV, FF, VS). The maximum iASPP expression within each TMA core was recorded. Nuclear expression was graded as absent (0), weak (1), moderate (2) or strong (3). Cytoplasmic expression was graded as absent (0), weak (1) or strong (2). iASPP expression in cells invading the prostatic capsule, or within intra-prostatic tumour, along with benign prostate epithelium in pT3 PCa whole-mount sections was scored in a similar manner by a histopathologist (CV).

### Statistical methods and patient survival analysis

Patient survival analysis was performed by Kaplan–Meier analysis using a log rank test and SPSS statistics 20 software (IBM, Portsmouth, UK). Paired and unpaired *t*-tests, and Mann–Whitney *U*-tests, were performed using GraphPad Prism (GraphPad, La Jolla, CA, USA) or excel software. All statistical tests were performed as two-tailed tests. Differences were considered significant at a *P*-value<0.05. All error bars shown on figures are S.E.M.

## Figures and Tables

**Table 1:** Clinical characteristics of the radical retropubic prostatectomy tissue microarray cohort

	**Radical retropubic prostatectomy** (***n*****=203)** ***n*** **(%)**
Median age (range)	63 (range 44–75) years
	
*Preoperative PSA*	
≤10	79 (38.9)
10.1–20	59 (29.1)
>20	64 (31.5)
Unknown	1
	
*pT-stage*	
≤pT2	94 (46.3)
≥pT3	109 (53.7)
	
*pN-stage*	
pN0	143 (70.4)
pN1	52 (25.6)
pN2	6 (3.0)
Unknown	2
	
*Gleason grade*	
≤6	137 (67.5)
7	36 (17.7)
≥8	29 (14.3)
Unknown	1
Median FU (range), years	8.8 (0.1–17.7)
Developed bone metastases at 10 years	43 (21.2)
Prostate cancer-specific death at 10 years	37 (18.2)

Abbreviations: FU, follow-up; PSA, prostate-specific antigen

**Table 2:** Multivariate analysis of clinical prognostic factors for the development of prostate cancer bone metastasis and prostate cancer-specific death in the radical retropubic prostatectomy tissue microarray cohort

	**Bone metastases**		**Prostate cancer death**	
	**HR (95% CI)**	***P***	**HR (95% CI)**	***P***
*All prostate cancer cases*				
pT stage	1.92 (0.96–3.85)	0.065	4.33 (1.75–10.69)	0.001
pN stage	1.58 (0.88–2.86)	0.129	1.96 (1.05–3.67)	0.035
PSA	1.01 (0.99–1.02)	0.505	1.00 (0.99–1.02)	0.828
Gleason group	2.32 (1.55–3.47)	<0.001	1.95 (1.23–3.10)	0.004
Nuclear iASPP expression	0.97 (0.47–1.98)	0.925	1.82 (0.91–3.66)	0.092
Cytoplasmic iASPP expression	0.98 (0.49–1.94)	0.948	1.26 (0.63–2.53)	0.508
				
*Gleason 7 prostate cancer cases*				
pT stage	6.36 (0.91–44.28)	0.062	3.72 (0.74–18.63)	0.110
pN stage	1.18 (0.31–4.54)	0.813	1.13 (0.36–3.60)	0.833
PSA	1.04 (1.0–1.09)	0.055	0.99 (0.94–1.04)	0.576
Nuclear iASPP expression	3.81 (0.80–18.1)	0.093	3.72 (1.10–12.61)	0.035
Cytoplasmic iASPP expression	0.56 (0.11–3.0)	0.501	0.53 (0.12–2.36)	0.403
				
*Gleason ≥8 prostate cancer cases*				
pT stage	3.84 (0.91–16.18)	0.066	8.20 (0.91–73.57)	0.06
pN stage	0.70 (0.19–2.53)	0.585	1.84 (0.46–7.33)	0.389
PSA	1.01 (0.99–1.03)	0.56	1.00 (0.98–1.02)	0.766
Nuclear iASPP expression	0.70 (0.16–3.13)	0.639	0.22 (0.03–1.47)	0.119
Cytoplasmic iASPP expression	4.58 (1.24–16.98)	0.023	8.17 (1.46–45.73)	0.017

Abbreviations: CI, confidence interval; HR, hazard ratio; iASPP, inhibitor of apoptosis-stimulating protein of p53; PSA, prostate-specific antigen

## Abbreviations

BrdU: bromodeoxyuridine
BSA: bovine serum albumin
DAPI: 4′,6-diamidino-2-phenylindole
DMEM: Dulbecco's Modified Eagle Medium
EDTA: ethylenediaminetetraacetic acid
EMT: epithelial-to-mesenchymal transition
FCS: Fetal calf serum
H&E: haematoxylin and eosin
HRP: horseradish peroxidase
iASPP: inhibitor of apoptosis-stimulating protein of p53
IF: immunofluorescence
IHC: immunohistochemistry
NET: 100 mM NaCl, 1 mM EDTA, 20 mM Tris
NHF: normal human fibroblast
PBS: phosphate buffered saline
PCa: prostate cancer
pT3: pathologic tumour stage 3
SDS: sodium dodecyl sulfate
TMA: tissue microarray
WB: western blotting
