# Supplementary figures and images for: Nuclear iASPP may facilitate prostate cancer progression
Source: Cell Death Dis. 2014 Oct 23;5(10):e1492–. doi: 10.1038/cddis.2014.442 (PMC4649527; doi:10.1038/cddis.2014.442)

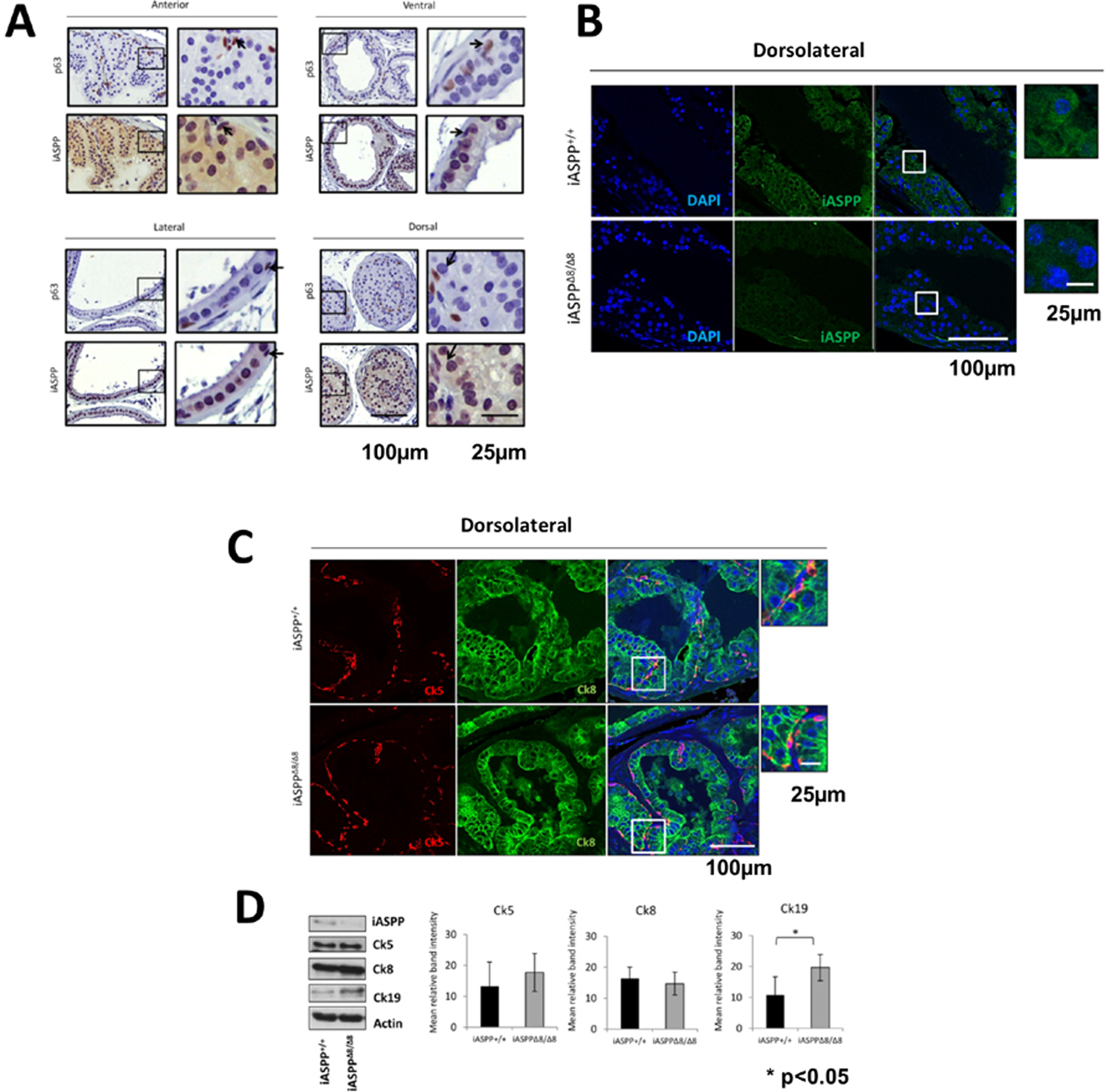

Supplement: Supplementary Figure 1 [file cddis2014442x1.tif]

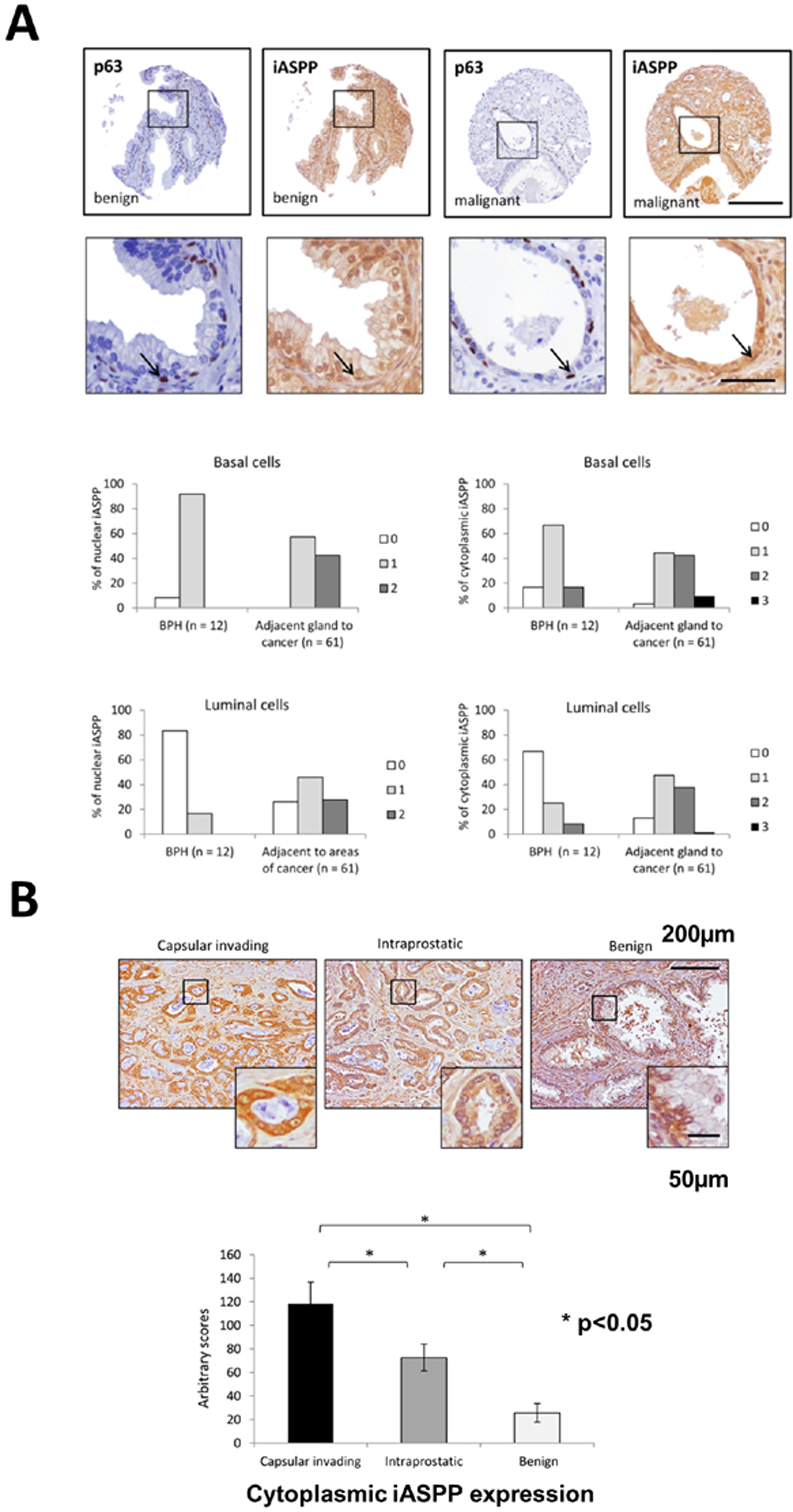

Supplement: Supplementary Figure 2 [file cddis2014442x2.tif]
